# Cancer Demographics and Time-to-Care in Belize

**DOI:** 10.1093/oncolo/oyad030

**Published:** 2023-03-16

**Authors:** Wayne Wong, James C Dickerson, Yannis K Valtis, Marta Habet, Margaret Bernard, Lorna Kelly, John Lattin, Philip Garrity, Rupali Sood, Alec Ohanian, Maryanne W Chege, Ami S Bhatt, Franklin W Huang, Ramon Yacab

**Affiliations:** Department of Medicine, University of Rochester School of Medicine and Dentistry, Rochester, NY, USA; Department of Medicine (Hematology and Oncology), Stanford University, Stanford, CA, USA; Department of Medicine, Brigham and Women’s Hospital, Boston, MA, USA; Medical Oncology, Karl Heusner Memorial Hospital, Belize City, Belize; Nursing Department, Karl Heusner Memorial Hospital, Belize City, Belize; Nursing Department, Karl Heusner Memorial Hospital, Belize City, Belize; Department of Medicine, Saint Louis University School of Medicine, St. Louis, MO, USA; Global Oncology, Inc., Oakland, CA, USA; Department of Medicine, Massachusetts General Hospital, Boston, MA, USA; Department of Medicine, UCSF School of Medicine, San Francisco, CA, USA; Department of Medicine, Stanford University School of Medicine, Palo Alto, CA, USA; Global Oncology, Inc., Oakland, CA, USA; Department of Medicine (Hematology, Blood and Marrow Transplantation) and of Genetics, Director of Global Oncology for the Stanford Center for Innovation in Global Health, Stanford University, Stanford, CA, USA; Global Oncology, Inc., Oakland, CA, USA; Division of Hematology and Oncology, Department of Medicine, San Francisco Veterans Affairs Medical Center, University of California San Francisco, San Francisco, CA, USA; Department of Urology, University of California San Francisco, San Francisco, CA, USA; Chan Zuckerberg Biohub, San Francisco, CA, USA; Medical Oncology, Karl Heusner Memorial Hospital, Belize City, Belize

**Keywords:** Caribbean, social determinants, delays, Belize, cancer

## Abstract

**Background:**

Belize is a middle-income Caribbean country with poorly described cancer epidemiology and no comprehensive cancer care capacity. In 2018, GO, Inc., a US-based NGO, partnered with the Ministry of Health and the national hospital in Belize City to create the first public oncology clinic in the country. Here, we report demographics from the clinic and describe time intervals to care milestones to allow for public health targeting of gaps.

**Patients and Methods:**

Using paper charts and a mobile health platform, we performed a retrospective chart review at the Karl Heusner Memorial Hospital (KHMH) clinic from 2018 to 2022.

**Results:**

During this time period, 465 patients with cancer presented to the clinic. Breast cancer (28%) and cervical cancer (12%) were most common. Most patients (68%) presented with stage 3 or 4 disease and were uninsured (78%) and unemployed (79%). Only 21% of patients ever started curative intent treatment. Median time from patient-reported symptoms to a biopsy or treatment was 130 and 189 days. For the most common cancer, breast, similar times were seen at 140 and 178 days. Time intervals at the clinic: <30 days from initial visit to biopsy (if not previously performed) and <30 days to starting chemotherapy.

**Conclusion:**

This study reports the first clinic-based cancer statistics for Belize. Many patients have months between symptom onset and treatment. In this setting, the clinic has built infrastructure allowing for minimal delays in care despite an underserved population. This further affirms the need for infrastructure investment and early detection programs to improve outcomes in Belize.

## Teaser

In 2018, the first public oncology clinic was established in Belize. This article reports demographics from the clinic and describes time intervals to care milestones to allow for public health targeting of gaps and provide a roadmap for public cancer care in the country.

**Implications for Practice**

This is the first report of cancer statistics from Belize; estimates from GLOBOCAN are drawn from Honduras and other neighboring countries. This is a critical first step toward understanding the burden of cancer in Belize. This article also describes stark delays in care; patients in Belize are taking a median of 4 months to get a biopsy after symptom development. This work identifies targets for public health interventions and future collaborative research. Lastly, it highlights the growth of the first and only public oncology clinic in Belize. This clinic has now treated hundreds of patients, mostly unemployed and uninsured, and provides a roadmap for public cancer care in the country.

## Background

Belize is located on the Caribbean coast of Central America and is classified by the World Bank as a low- and middle-­income country (LMIC).^1^ It has limited cancer demographic data and poor cancer care infrastructure. Government funding for healthcare is at 3.5% of GDP, below the Pan American Health Organization/World Health Organization (PAHO/WHO) recommended minimum of 6%.^,2-4^ Few resources have historically been dedicated to oncologic care; treatment is costly and largely paid for out-of-pocket.^3^ In 2022, the populous of over 400,000 has 2 diagnostic pathologists and a single medical oncologist; radiotherapy capabilities are not available in-nation and general surgeons perform oncologic surgeries.^4-6^ Many patients who can afford to travel choose to go to Mexico and Guatemala for treatment.

In 2018, Global Oncology, Inc. (GO), a US-based non-profit organization with the mission to improve cancer care in LMICs, partnered with Karl Heusner Memorial Hospital (KHMH) in Belize City and the Belizean Ministry of Health (MoH) to develop the first public oncology clinic in the country^.7^ Alongside capacity building, there were 2 research aims that were identified by the partnership. These aims were chosen based on MoH’s noncommunicable disease strategic action plan.^3^ The aims were (1) to generate the first cancer demographics for the nation and (2) to quantify gaps in care. Gaps were quantified using previously reported metrics of the time from symptom onset to key oncology care milestones (eg, biopsy, first visit, etc.).^8^ This was done for all patients at the clinic, and the most common cancer, breast. We also focus on the 2 most prevalent cancers in Belize, breast and cervical, given cancer control of breast and cervical cancer are priorities of the MoH. Here we report on these research aims, discuss the partnership, and outline future work.

## Patients and Methods

### IRB/Approvals/Data Security

Prior to study initiation, cancer treatment protocols were reviewed and discussed with all stakeholders. KHMH does not have an Institutional Review Board, and so the protocol was reviewed by the University of Rochester Institutional Review Board. It was also provided to relevant KHMH administrators for review and feedback. A waiver of informed consent was granted to abstract patient health information. Approvals from both institutions were obtained before data collection began. Privacy and confidentiality were maintained via a secure record system on site at the clinic. All de-identified data was stored on a secure password-protected cloud storage system hosted by the University of Rochester (Box, Redwood City, CA).

### Study Outline

Patients seen at the KHMH oncology clinic between 2018 and 2022 were included in the retrospective chart review. The KHMH oncology clinic collects and stores detailed cancer care-specific data in the CommCare mobile platform (Dimagi, Inc., Cambridge, MA). This system runs parallel to patient charts.^9^ We used this platform, implemented in 2020, as the primary data set. Data from patients seen prior to the use of CommCare were manually entered from paper charts. Each patient seen after 2020 was cross-referenced with available paper charts to ensure accuracy. Lastly, as an additional step to ensure accuracy, charts were referenced against the national Belizean Health Information System (BHIS). The BHIS stores basic demographic information about patients and is not universally used by clinicians.^10^ Data were abstracted from February to April 2022. Demographic indicators examined included age, ethnicity, education level, and employment status; clinical factors such as cancer type, stage at presentation, and treatment intent were also collected. For the breast cancer population, further review of paper charts supplemented by the CommCare data allowed for additional information, such as histology and immunohistochemistry profiles, to be captured (see supplement for full list). We defined lost to follow up as no available records in any system (CommCare, paper chart, or BHIS) for > 6 months.

Time from symptom development to various care milestones (biopsy, oncology clinic visit, chemotherapy, upfront surgery) is referred to as time-to-care. For the time-to-care analyses, only patients with available treatment data were included. Relevant dates were captured by locating individual clinical notes documenting initial presentation to the KHMH oncology clinic, pathology reports, chemotherapy orders, and surgical reports in CommCare and manually cross-referenced with available paper charts and BHIS. Time-to-care was measured by calculating elapsed time between symptom onset for a particular patient and documented cancer care milestones, consisting of date of first presentation to the KHMH oncology clinic, date of biopsy result, date of initial chemotherapy infusion, and surgery. Symptom onset was patient reported and consistently documented by the treating physician (RY) both in paper charts as well as in the research database where it was a discrete field collected intentionally for this metric. We excluded surgeries that occurred after neo-adjuvant chemotherapy from the time-to-care analyses.

Baseline demographic information, clinical characteristics, and time-to-care milestones were summarized using descriptive statistics. Categorical data were described in frequencies and percentages. Continuous variables were summarized as medians with interquartile ranges (IQR). Data analysis was conducted in Microsoft Excel 365 (Microsoft Inc., Redmond, WA).

## Results

Details of 533 patients were gathered from the opening of the clinic in April 2018 until April 2022. Four hundred and twenty-two patients were registered in CommCare, with an additional 111 patients added after review of 351 available paper charts. Many abstracted demographic and clinical variables such as patient ethnicity and level of education were not consistently captured during the early clinic period and during the initial implementation of CommCare. Data are missing for some patients, therefore a sample size is given for each variable.

### Patient Demographics

**Figure 1.**
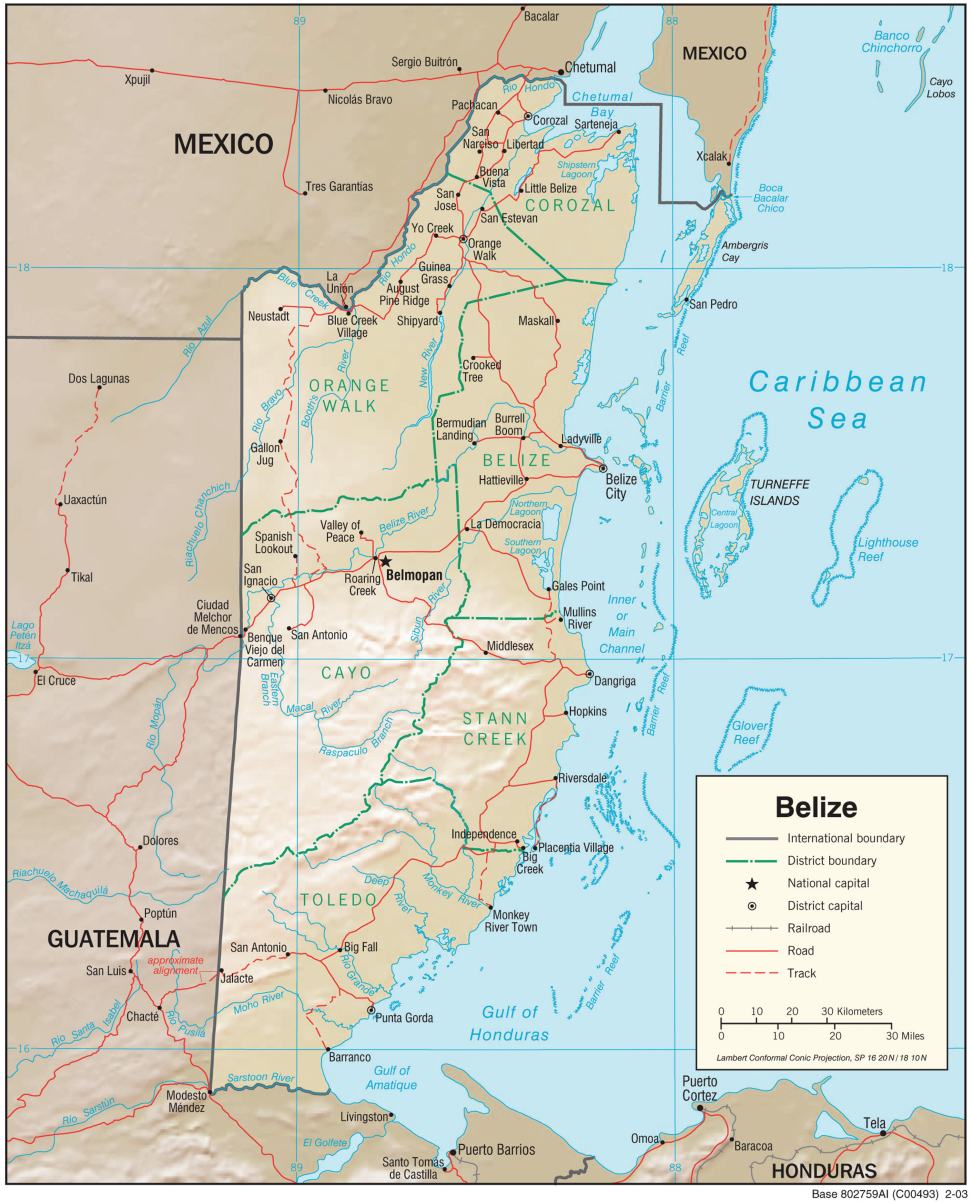
Map of Belize and its districts. Patients seen in the clinic came from all the districts of Belize, though predominantly from those close to the clinic and with transportation infrastructure. Percentages were as follows: Belize district (44%), Cayo (19%), Orange Walk (14%), Corozal (11%), Stann Creek (8%), and Toledo (5%). Map courtesy of the University of Texas Libraries, The University of Texas at Austin.

Socioeconomic and demographic variables are shown in Table 1. The median age was 55 years with a broad range (2-95 years). Patients were largely unemployed (78%) and without health insurance (79%). Of the 21% of patients with insurance, almost all (63 of 68) were covered by the Belizean government National Health Insurance (NHI). Regarding patients’ home districts, the majority (57%) came from outside of Belize District where the clinic is located (Fig. 1).

**Table 1.** Socioeconomic demographics of cancer patients at the KHMH oncology clinic in Belize City, Belize.

Characteristic	Clinic population (*n* = 533)
Age in years, median (IQR), *n* = 532	55 (42-65)
Sex, no. (%), *n* = 497	
Male	152 (31%)
Female	345 (69%)
Ethnicity, no. (%), *n* = 456	
Creole	182 (40%)
Mestizo	180 (40%)
Hispanic/Spanish	29 (6%)
Garifuna	18 (4%)
East Indian	14 (3%)
Maya	13 (3%)
White	8 (2%)
Mennonite	5 (1%)
East Asian	1 (<1%)
Other	4 (1%)
Unknown	2 (<1%)
Districts of residence, no. (%), *n* = 442	
Belize	195 (44%)
Cayo	82 (19%)
Orange Walk	61 (14%)
Corozal	47 (11%)
Stann Creek	37 (8%)
Toledo	20 (5%)
Education, no. (%), *n* = 329	
Primary	165 (50%)
Secondary	74 (23%)
Tertiary	29 (9%)
Other	13 (4%)
None	22 (7%)
Unknown	26 (8%)
Employment, no. (%), *n* = 418	
Employed	87 (21%)
Unemployed	324 (78%)
Prefer not to answer	7 (2%)
Health insurance, no. (%), *n* = 327	
Yes^*^	68 (21%)
No	259 (79%)

This table shows the demographic characteristics of the clinic and are reflective of the diverse nature of Belize. To provide context for a few points, there are no clear statistics on what percentage of Belizean citizens are insured (either privately or publicly). The unemployment rate in our population is high. Belize reports an unemployment rate no greater than 12% for the nation over the time of data collection. However, looking at a September 2021 government report as an example, the reported unemployment rate excludes 38% of the working-age population as they were “outside the labor force.” For women, this report advances that they are outside the labor force due to “personal or family responsibilities.” Thus, the high unemployment rate in the clinic (in comparison to World Bank estimates and government statistics) likely reflect different methodologies of reporting, though disability due to cancer likely accounts for a percentage as well.

^*^Sixty-three of 68 (93%) with health insurance had the Belize National Health Insurance plan. Two patients (3%) reported having private insurance and another 3 persons (4%) did not specify their insurer.

### Clinical Characteristics

**Figure 2.**
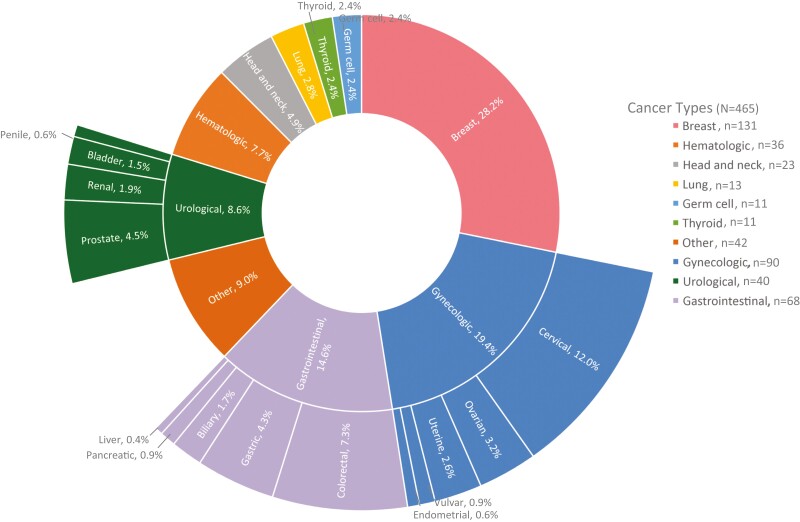
Cancer types seen at KHMH. Here we report the cancer types seen at the clinic. GLOBOCAN’s 2020 report uses neighboring nations to estimate statistics for Belize. In comparison to the GLOBOCAN estimates, the clinic saw more breast patients (28% at the clinic versus 22% based on estimates by GLOBOCAN), less prostate cancer (5% versus 20% estimate), and less liver cancer (<1% versus 8.7% estimate). Other cancers were similar to the estimates in this GLOBOCAN report.

The clinical characteristics and cancer types are shown in Fig. 2; Table 2. Diagnoses were made using H&E staining. Immunohistochemistry (IHC) is unavailable in Belize. Patients who can afford IHC coordinate shipment of their specimens to out-of-country pathology labs. The most common cancers observed (*n* = 465) were breast (28%), cervical (12%), hematologic malignancies (8%), colorectal (7%), head and neck cancers (5%), and prostate (4%). Of the 352 patients able to be clinically staged using the American Joint Committee on Cancer (AJCC) 7th edition guidelines, 68% of patients had stage 3 (*n* = 97, 28%) or stage 4 (*n* = 142, 40%) disease at presentation.^11^ A notable portion (20%) of patients remain with the status of under diagnostic work-up, indicating additional diagnostic studies are needed to determine treatment intent. While 54% of this is data censoring at the time of abstraction, 46% (42 of 90) of these “work-up” patients were lost to follow up.

**Table 2.** Clinical characteristics of KHMH oncology clinic cancer patients.

Characteristic	Clinic population (*N* = 533)
Pathologic diagnosis, no. (%), *n* = 465	
Confirmed	380 (82%)
Suspected	85 (18%)
Clinical stage, no. (%), *n* = 352	
Stage 0	1 (<1%)
Stage I	25 (7%)
Stage II	87 (25%)
Stage III	97 (28%)
Stage IV	142 (40%)
Treatment intent*, no. (%), *n* = 460	
Curative	97 (21%)
Palliative	105 (23%)
Best supportive care**	74 (16%)
Surveillance^†^	94 (20%)
Diagnostic workup^‡^	90 (20%)

^*^Initial treatment intent as recorded by the treating physician (RY) after the completion of work-up. Forty-six percent of the in the diagnostic work-up category were lost to follow up **Medical management of cancer symptoms including palliative care (pain, dyspnea management, etc.) without the use of antineoplastic agents. There is limited palliative infrastructure in Belize.

^†^Regularly scheduled clinic follow-up only, typically every 6 months to 1 year

^‡^Fifty-four percent (48 of 90) of patients remain in this category due to data censoring at the time of abstraction. The other 46% had no clinic follow-up nor records for at least 6 months and thus were considered lost to follow up.

### Time-to-Care Analysis

**Figure 3.**
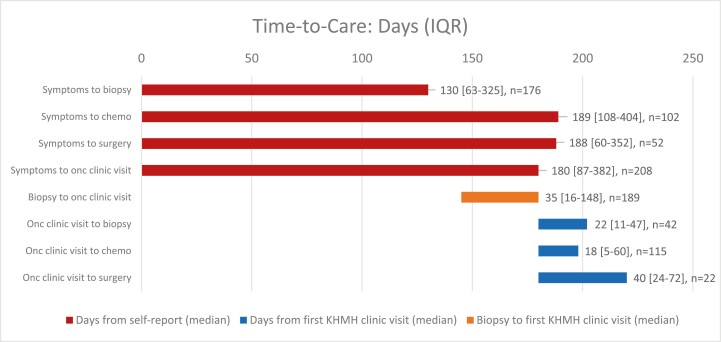
Time-to-care intervals. Bars indicate time from patient-reported symptom development to a care milestone such as chemotherapy or surgery. This graph includes patients who received treatment at any location (ex. Belize, Mexico, Guatemala). Time of symptom development was patient-reported and consistently recorded by the treating physician (RY) at the initial clinic visit. Surgery following neo-adjuvant chemotherapy is excluded from the above figure. While we have dates for biopsies, surgeries, and chemotherapy, we were unable to identify time from symptom to any medical contact. Patients seeking care had long delays from symptom development till care milestones (oncology clinic visit, biopsy, starting treatment). Future work will explore why these delays occurred. Patients received cancer care expeditiously (<60 days) once seen at the KHMH oncology clinic, showing infrastructure can be developed to deliver timely care.

We calculated time intervals for select cancer care milestones, shown in Fig. 3. For chemotherapy at any site (ex. Belize, Mexico, Guatemala), the median number of days between self-reported symptom onset and treatment initiation was 189 days (IQR 108-404). For surgery at any site (ex. Belize, Mexico, Guatemala), median number was 188 days (IQR60-352). Patients who sought initial cancer care at KHMH presented after a median of 180 days (IQR 87-382 days). This excluded patients (*n* = 84) who transferred to KHMH after first seeking out-of-country oncology care. To examine clinic performance, we also examined milestones occurring after the initial clinic visit at KHMH. These are listed in blue in Fig. 3.

### Breast Cancer Patient Demographics and Clinical Characteristics

Breast cancer, the most common cancer worldwide, was the most common cancer seen at the clinic.^2^ Given this, we further examined the clinical characteristics of this group. Demographics and clinical characteristics were similar to the overall population reported in Table 1 and are available in Supplementary Table S4.

### Histologies, IHC, and Treatment of Breast Cancer Patients

Available data on histology and IHC profiles can be found in Table 3. Of the patient with breast cancer, 44 of 131 (34%) self-coordinated sending their specimens for IHC testing out-of-country (>50% of the IHC was performed in the US while other specimens were sent to Mexico, Guatemala, and El Salvador). Patients without available IHC were treated as estrogen receptor positive, typically with neoadjuvant chemotherapy and post-operative endocrine therapy. Cytotoxic chemotherapies in the curative setting were either taxane or anthracycline based. Most breast cancer patients (*n* = 87, 66%) received chemotherapy. One patient was able to procure trastuzumab privately. Half of the breast cancer patients (65 of 131) received surgery, typically performed in Belize, Guatemala, or Mexico. Of patients who had their procedures described (59 of 65), surgery most often consisted of modified radical mastectomy with lymph node dissection (52 of 59 surgeries). In Belize, these were performed by general surgeons (*n* = 45), and of those who received a mastectomy outside of Belize, 2 patients traveled to the US, 4 to Mexico, and 1 to Guatemala. Breast conserving surgeries were rare (7 of 59 surgeries). Less than a quarter (22 of 131, 17%) of patients received adjuvant radiotherapy. All of these patients traveled to either Mexico or Guatemala for radiation. A little over a quarter (*n* = 35, 27%) of all breast cancer patients had documented use of endocrine therapy, about a 50:50 split of aromatase inhibitors and tamoxifen. A significant proportion of patients (20%; 38 of 131) did not have any treatment information available.

**Table 3.** Clinical characteristics of KHMH breast cancer patients.

Characteristic	Breast cancer population (*n* = 131)
Clinical stage, no. (%), *n* = 114	
Stage 0	1 (1%)
Stage I	3 (3%)
Stage II	44 (39%)
Stage III	46 (40%)
Stage IV	20 (18%)
Treatment intent*, no. (%), *n* = 130	
Curative	41 (32%)
Palliative	21 (16%)
Best supportive care^**^	6 (5%)
Surveillance^†^	44 (34%)
Diagnostic workup^‡^	18 (14%)
Any treatment received, no. (%), *n* = 131	
Chemotherapy	87 (66%)
Surgery	65 (50%)
Radiotherapy	22 (17%)
No treatment noted	38 (29%)
Breast cancer histology, no. (%), *n* = 92	
Invasive ductal carcinoma	71 (77%)
Invasive lobular carcinoma	5 (5%)
Papillary carcinoma	4 (4%)
Medullary carcinoma	3 (3%)
Inflammatory	1 (1%)
Other	8 (9%)
ER/PR/HER2 profiles, no. (%), *n* = 44	
ER+	33 (75%)
PR+	29 (66%)
HER+	6 (14%)
Triple Negative	9 (20%)
HR+/HER2−	26 (59%)
HR+/HER2+	6 (14%)
HR-/HER2+	2 (4%)
Triple Negative	10 (23%)

The socioeconomic and demographics of the breast cancer patients at KHMH were similar to the overall cohort’s reported in Table 1. Staging recorded here is clinical. Depending on what patients could afford, imaging was done either with CT scans, or, a combination of x-rays and a liver ultrasound.

^*^Treatment intent at the time of the initial work-up/ staging having been completed.

^**^Medical management of cancer symptoms including pain and dyspnea without the use of antineoplastic agents.

^†^Regularly scheduled clinic follow-up only, typically every 6 months to 1 year.

^‡^54% (48 of 90) of patients remain in this category due to data censoring at the time of abstraction. The other 46% had no clinic follow-up nor records for at least 6 months and thus were considered lost to follow up.

**Figure 4.**
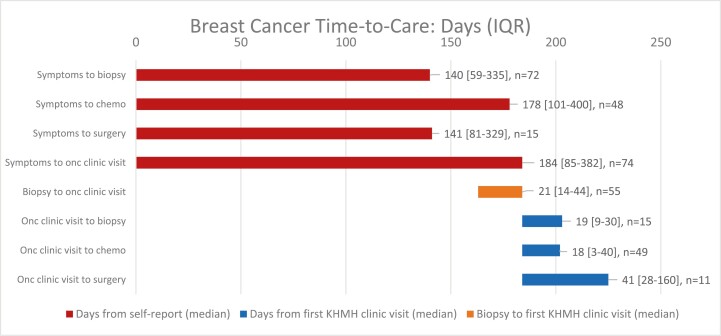
Time-to-care for breast cancer patients. Bars indicate time from patient-reported symptom development to a care milestone such as chemotherapy or surgery. This graph includes patients who received treatment at any location (ex. Belize, Mexico, Guatemala). Time of symptom development was patient-reported and consistently recorded by the treating physician (RY) at the initial clinic visit. Surgery following neo-adjuvant chemotherapy is excluded from the above figure. Data on time to first medical contact was not available. While the time to KHMH was long, once seen at the clinic care occurred relatively quickly (<60 days) demonstrating successful infrastructure.

Time-to-care intervals for breast cancer patients are shown in Fig. 4. The time-to-surgery is for upfront surgical management only.

### Cervical Cancer Patient Demographics and Clinical Characteristics

The Ministry of Health has made efforts to enact a national cervical cancer control program in response to a focus on non-communicable diseases in its most recent Belize National Health Sector Strategic Plan (2014-2024). Due to this national priority, we provide a brief overview of cervical cancer at the KHMH clinic. Cervical cancer was the second most common cancer seen at the clinic, making up 12% (*n* = 56 of 465) of all patients and 62% (*n* = 56 of 90) of patients with gynecologic cancers. These women with cervical cancer presented at a median age of 50 (IQR 42-59). Few (*n* = 2, 4%) presented with stage 1 disease. The majority presented with stage 2 (*n* = 21, 39%), stage 3 (*n* = 16, 30%), and stage 4 (*n* = 12, 22%) disease.

## Discussion

In this retrospective cohort study, we describe patient characteristics and time intervals to care for cancer patients at the first and only public oncology clinic in Belize. We found most patients presented with late-stage disease and had large delays of 4-6 months in obtaining biopsies, starting chemotherapy, and having surgeries done after symptoms developed. In contrast to these long times, once patients were established at the KHMH clinic the time-to-therapy was relatively short. This highlights that successful care infrastructure can be built in the nation.

### Stage at Presentation

Patients presented predominantly with stage 3 (*n* = 97, 28%) or stage 4 (*n* = 142, 40%) (combined *n* = 239, 68%).^12^ For breast cancer, the rate (58%) of stage 3 or 4 disease was higher than prior reported ranges (37%-45%) in Central America.^13^ We suspect that the drivers of these late-stage presentations are multi-factorial, though dominated by social determinants of health.^14^ This patient population was largely unemployed (78%) and most patients came from outside Belize City. They were likely traveling between 1 and 7 h each way for visits. Most patients were not insured, and those that did have insurance were usually covered by the NHI. The NHI government insurance program is only available to patients living in impoverished areas (southern Belize City, the southern Districts, and Corozal) and only covers basic diagnostic services and primary care.^4^ Even for the insured in this study, there were significant out-of-pocket costs. While some services at the cancer clinic are provided for free, patients do have to pay for chemotherapy and scans (including NHI insured patients). Statistics on how many Belizeans are insured are not readily accessible, though it is not likely for most citizens. Patients at the KHMH clinic reported funding their care through a mix of methods: having community fundraisers, selling home-cooked meals, and obtaining loans. Future capacity building must consider the costs incurred by patients.

### Time-to-Care

The median time from symptom onset to treatment initiation was 6 months (chemotherapy: 189 days [IQR 108-404]; surgery: 188 days [IQR 60-352]). Several studies report worse outcomes with treatment delays.^15,16^ While we do not have outcome data for this cohort of patients, we think that trying to reduce the delays to increase the fraction of patients who can undergo curative intent treatment is high value investment for cancer care.

Breast cancer patients at the clinic also experienced substantial delays in presentation, with a median time-to-care of 170 days (IQR 98-379). This delay is consistent with past observations from the Caribbean. In a 2009 survey of Caribbean oncologists (not including Belize), 30% of the clinicians reported an average of >90 days from symptom onset to imaging or a clinical exam.^17^ A decade after this 2009 report, Belize still shows these significant delays. There continue to be opportunities for improvement of Caribbean cancer care.

### Partnership

The partnership between the Belizean MoH, the KHMH clinic, and GO enabled the rapid development of a robust clinic infrastructure. The clinic is small: 4 nurses (2 oncology nurses and 2 patient navigators), a pharmacist, and the only medical oncologist in Belize. Despite being in its infancy (a soft launch in spring 2018, and an official launch in 2021) by 2022 the clinic had seen at least 533 cancer patients since inception, with year-over-year increases in patient volume.^18^ We highlight the relatively short times from the initial visit to care initiation at the clinic, in comparison to the background rate (Fig. 3). Patients at the clinic receive care within recommended timelines for high income countries.^19^ This shows that building this infrastructure for complex cancer care is feasible in Caribbean LMICs, even without fully addressing many of the societal barriers (cost, travel).

The care capacity was made possible by bi-directional partnerships between the MoH, KHMH, and GO resulting in the development of safe treatment protocols, training of nurses and patient navigators, and government funding. One of the clinic’s successes has been to procure a regular stock of chemotherapy medications in partnership with the NHI. Due to the absence of a chemotherapy supply chain prior to 2021, patients treated prior to 2021 acquired chemotherapy outside of Belize and brought it back to the clinic for administration. While the cost of chemotherapy remains a barrier today, the supply chain was the only way for Belizeans to get chemotherapy during border closures with Mexico and Guatemala during the COVID-19 pandemic.

### Belize Cancer Registry and National Cancer Control Strategies

There is no cancer registry or screening program in Belize today. This partnership showed that data collection to inform policy and research is feasible without being overly burdensome to clinic staff. A cancer registry could be built out from the clinic’s system, though would need to be interoperable with the private sector, and ideally would be directly built out of BHIS rather than a separate system. While work on a registry dates back to a 2008 collaboration between the Belize Cancer Society and the MoH, there is renewed energy in developing a comprehensive registry in 2023.

We wish to highlight efforts aimed at earlier detection and prevention of cancer in Belize. Non-communicable diseases are now the most common causes of death in the nation.^20^ In 2016 the MoH in partnership with the non-profit Jhpiego developed the Cervical Cancer Prevention and Control Strategic Plan (2016-2020).^21^This plan was a follow-up to the MoH’s focus on noncommunicable diseases in the 2014 Belize National Health Sector Strategic Plan. Human papillomavirus vaccinations were integrated into the national vaccine schedule by MoH as part of the country’s primary prevention strategy, and secondary and tertiary prevention services for screening, early detection, and treatment of pre-cancerous and cancerous lesions were expanded. These national efforts are timely—our data showed cervical cancer to be the second most common malignancy overall at the clinic with stages 2 and 3 diseases being the most common stages at presentation.

The data on time-to-care have yielded new goals and research aims—specifically to understand how these delays arise and how to reduce them. While for cervical cancer the MoH strategy centers around vaccination and screening, for breast cancer, understanding the delays and how to get symptomatic patients to treatment quickly are next steps. The goal is to downstage the presentations and increase the curative fraction.

### Limitations

This study has several limitations: it is a retrospective chart review of a single oncology clinic. There is a lack of a unified health system to ensure accurate reporting, and we relied on patient reporting and primarily internal data. We lack any outcome data. Despite these limitations, this is the first data set on cancer patients in the nation and provides a starting point for improving cancer care in Belize.

## Conclusions

This study describes cancer demographics and quantifies delays in care experienced in a cohort of 533 patients with cancer at the only public oncology clinic in Belize. Our data show Belizean patients experience lengthy delays in all aspects of cancer care. The financial toxicities of the diagnostic work-up, and of longitudinal cancer care, likely play a large role in these delays. Additionally, anecdotal accounts provided by patients and clinic staff suggest low cancer literacy, lack of awareness among primary care providers of the new public oncology clinic in Belize and hence lack of referral, and sociocultural beliefs potentially hindering discussions of cancer and available treatments in Belize are additional factors in these delays. Further research is needed to qualify and quantify these barriers. Continued allocation of resources to high-value interventions such as HPV vaccinations and acetic acid cervical cancer screening, and studying where care systems break down will impact outcomes.^22^ This work shows that cancer care infrastructure can be built in Caribbean LMICs. These are encouraging starts to an investment in non-communicable disease infrastructure in Belize.

## Supplementary Material

oyad030_suppl_Supplementary_Material

## Data Availability

The data underlying this article will be shared on reasonable request to the corresponding author.

## Prior Presentation

This work was presented as an abstract at ASCO 2022.
