# Supplementary material for: Cancer Demographics and Time-to-Care in Belize
Source: Oncologist. 2023 Mar 16;28(6):e350–8. doi: 10.1093/oncolo/oyad030 (PMC10243772; doi:10.1093/oncolo/oyad030)
Supplement: oyad030_suppl_Supplementary_Material [file oyad030_suppl_supplementary_material.docx]

**Supplement**

**Table of Contents**

Page 2 – 3: Supplementary tables

Pages 4 – 7: List of all recorded variables in CommCare

| **Characteristic** | **Clinic Population (N = 533)** |
| --- | --- |
| Cancer histologies, No. (%), n = 465 |  |
| Breast | 131 (28%) |
| Cervical | 56 (12%) |
| Hematologic | 36 (8%) |
| Colorectal | 34 (7%) |
| Head and neck | 23 (5%) |
| Prostate | 21 (5%) |
| Gastric | 20 (4%) |
| Ovarian | 15 (3%) |
| Lung | 13 (3%) |
| Uterine | 12 (3%) |
| Germ cell | 11 (2%) |
| Thyroid | 11 (2%) |
| Renal | 9 (2%) |
| Sarcoma (Other) | 9 (2%) |
| Biliary | 8 (2%) |
| Bladder | 7 (2%) |
| Bone (Other) | 7 (2%) |
| CNS (Other) | 5 (1%) |
| Pancreatic | 4 (1%) |
| Vulvar | 4 (1%) |
| Endometrial | 3 (1%) |
| Penile | 3 (1%) |
| Liver | 2 (<1%) |
| Cutaneous (Other) | 2 (<1%) |

**Table format of figure 2. Cancer histologies seen at KHMH.**

| **Characteristic** | **Breast cancer population (n= 131)** |
| --- | --- |
| Age in years, median (IQR), n = 131 | 53 (46 - 62) |
| Sex, No. (%), n = 131 |  |
| Male | 2 (2%) |
| Female | 129 (98%) |
| Education, No. (%), n = 80 |  |
| Primary | 40 (50%) |
| Secondary | 26 (33%) |
| Tertiary | 11 (14%) |
| None | 3 (4%) |
| Employment, No. (%), n = 100 |  |
| Employed | 15 (15%) |
| Unemployed | 83 (83%) |
| Prefer not to answer | 2 (2%) |
| Health Insurance, No. (%), n = 91 |  |
| Yes | 21 (23%) |
| No | 70 (77%) |
| If “Yes” to Health Insurance, Type, No. (%), n = 21 |  |
| NHI | 20 (95%) |
| Private | 1 (5%) |

**Table 4. Socioeconomic demographics of breast cancer patients at KHMH.** These data are similar to the overall cohorts and complements table 3 in the paper

**Data dictionary of all variables recorded in CommCare system for all patients and breast cancer patients:**

All patients:

- First Name
- Last Name
- BHIS
- DOB (MM/DD/YYYY)
- Calculated age at first visit
- Gender
- Race
- District
- City/Town/Village
- Does patient have a secondary language?
- Primary language
- Other primary language
- Employment
- Health insurance
- Types of health insurance
- Education level
- Religious
- Religion
- Date of first consult
- Consultation site
- Current ECOG score
- Cancer status
- Diagnostic status
- Anatomic stage
- Clinical stage
- Oncologic category
- Diagnosis
- Treatment intent
- Clinical status
- Treatment type
- Type of chemo
- Know when symptoms began
- Symptom start date
- Pcp referral
- Pcp referral date
- Biopsy prior
- Date of biopsy result
- HER2 status
- Hormone receptor status
- Staging CT completed
- Staging CT date
- cTNM
- Status tumor marker
- Chemo prior
- First chemo infusion date at KHMH
- Other chemo infusion location
- Regimen line
- Regimen codename
- Other regimen
- Planned number of cycles
- Surgery prior
- Surgery date
- Surgery prior description
- Surgical procedure
- radiotherapy performed
- radiotherapy month
- radiotherapy procedure

Breast cancer patient variables:

- First Name
- Last Name
- BHIS
- DOB (MM/DD/YYYY)
- Calculated age at first visit
- Gender
- Oncologic category
- BMI
- Registration age
- Preferred language
- Place of Residence
- District
- Health Insurance (Y/N)
- Type of health insurance
- Nationality
- Religion
- Ethnicity
- Education
- Employment (Y/N)
- Date of first sxs - self report
- First sxs - self report
- PE at clinic presentation
- Menopause?
- PCP referral date
- Biopsy?
- Biopsy date
- Biopsy result date
- Diagnosis
- Grade
- IHC
- reflex to FISH?
- IHC lab
- First consult date
- ECOG
- Staging CT?
- CT dates
- Dx status
- Cancer status
- Anatomic stage
- cTNM
- Clinical stage
- Clinical status
- Treatment intent
- Chemo type
- Chemo regimen prescribed at diagnosis
- Cycles completed (initial regimen)
- Alternate chemo regimen
- Cycles completed (alternate regimen)
- Reason for change
- First neoadjuvant infusion date
- Subsequent neoadjuvant infusion dates
- Neoadjuvant response
- Surgery date
- Surgery procedure
- First consult s/p surgery
- First adjuvant infusion date
- Subsequent adjuvant infusion dates
- XRT date
- Hormone treatment
- Last date of follow up
- Any treatment for previous breast cancer
- First consult s/p recurrence
- First palliative infusion date
- Subsequent palliative infusion dates
